# Epigenetic regulation of major histocompatibility complexes in gastrointestinal malignancies and the potential for clinical interception

**DOI:** 10.1186/s13148-024-01698-8

**Published:** 2024-06-24

**Authors:** Jorge Enrique Tovar Perez, Shilan Zhang, William Hodgeman, Sabeeta Kapoor, Praveen Rajendran, Koichi S. Kobayashi, Roderick H. Dashwood

**Affiliations:** 1 Center for Epigenetics and Disease Prevention, Texas A&M Health, Houston, TX 77030 USA; 2 Department of Cardiovascular Medicine, Shanghai Tenth People’s Hospital, Tongji University School of Medicine, Shanghai, 200070 China; 3 Wolfson Medical School, The University of Glasgow, Glasgow, G12 8QQ UK; 4 Department of Translational Medical Sciences, and Antibody & Biopharmaceuticals Core, Texas A&M Medicine, Houston, TX 77030 USA; 5 Department of Immunology, Hokkaido University Graduate School of Medicine, Sapporo, 060-8638 Japan; 6 Hokkaido University Institute for Vaccine Research and Development, Sapporo, 060-8638 Japan; 7 Department of Microbial Pathogenesis and Immunology, Texas A&M Health, Bryan, TX 77087 USA

**Keywords:** Gastrointestinal cancer, Epigenetic regulation, MHC, Immunotherapy, Cancer immune evasion

## Abstract

**Background:**

Gastrointestinal malignancies encompass a diverse group of cancers that pose significant challenges to global health. The major histocompatibility complex (MHC) plays a pivotal role in immune surveillance, orchestrating the recognition and elimination of tumor cells by the immune system. However, the intricate regulation of MHC gene expression is susceptible to dynamic epigenetic modification, which can influence functionality and pathological outcomes.

**Main body:**

By understanding the epigenetic alterations that drive MHC downregulation, insights are gained into the molecular mechanisms underlying immune escape, tumor progression, and immunotherapy resistance. This systematic review examines the current literature on epigenetic mechanisms that contribute to MHC deregulation in esophageal, gastric, pancreatic, hepatic and colorectal malignancies. Potential clinical implications are discussed of targeting aberrant epigenetic modifications to restore MHC expression and 0 the effectiveness of immunotherapeutic interventions.

**Conclusion:**

The integration of epigenetic-targeted therapies with immunotherapies holds great potential for improving clinical outcomes in patients with gastrointestinal malignancies and represents a compelling avenue for future research and therapeutic development.

## Background

Our understanding of epigenetic regulation in physiology and pathophysiology is constantly evolving. Reversible DNA methylation signatures and post-translational modifications to histones, such as methylation, acetylation, phosphorylation, and ubiquitination, play pivotal roles in development and cellular homeostasis. Epigenetic modifications can activate or suppress gene expression in response to both intrinsic and extrinsic stimuli. The counterpoint to this yin/yang arrangement is that inappropriate gene expression can be associated with common pathologies, including cancer etiology. Hypermethylation in gene promoter regions, genome-wide hypomethylation, and histone deacetylation are hallmarks of aberrant gene transcription in various malignancies [1]. A deeper appreciation of tissue-specific epigenetic regulation, with a view to enhanced mechanistic targeting and precision oncology, will open new avenues for cancer interception and novel therapeutic approaches.

Recent advances also have highlighted the crosstalk between epigenetic regulation and the immune system. Major histocompatibility complexes class I and class II (MHC-I and MHC-II) are key components of the adaptive immune response. These human glycoproteins interact with specific pathogenic antigens, resulting in cell surface T cell recognition, and engagement of the host immune system. Epigenetic mechanisms have critical roles in the regulation of MHC-I- and MHC-II-associated genes, the corresponding enhanceosomes, and the master transactivators [2].

Because of their central roles in adaptive immunity, MHC complexes have received interest in the context of immune responses to metastasis and epigenetic regulation. Ideally, antigen-specific CD8^+^ and CD4^+^ T cells respond, respectively, to MHC-I and MHC-II cell surface complexes on cancer cells, activating host immune mechanisms that target the tumor for removal [2]. However, altered MHC expression due to malignant transformation and epigenetic deregulation can dampen tumor detection and engagement of the adaptive immune response [2, 3]. The counterpoint, however, is that clinical cancer interception and re-engagement of host immunity are viable strategies arising from the reversibility of epigenetic landscapes in precancer and cancer stages.

We sought to provide an overview of the epigenetic deregulation of MHC-associated factors in the pathogenesis of intestinal, hepatic, and pancreatic malignancies, broadly classified herein as gastrointestinal (GI) cancers. The latter are characterized by epigenetic silencing and concomitant alterations in gene expression that appear in distinct gut pathologies [3]. It is known that GI tumors lack MHC-I and MHC-II expression, which contributes to low CD8^+^ and CD4^+^ T cell tumor infiltration and poor prognosis. Thus, new therapeutic approaches that upregulate MHC expression could result in enhanced patient outcomes [4, 5]. Epigenetic landscapes of GI tumors are of current scientific interest with a view to understanding immune evasion in the gut, and to guide future development of novel cancer immunotherapies and immunopreventive approaches. These aspects will be discussed in the following sections.

## Epigenetic regulation of MHC-I and MHC-II

**Fig. 1:**
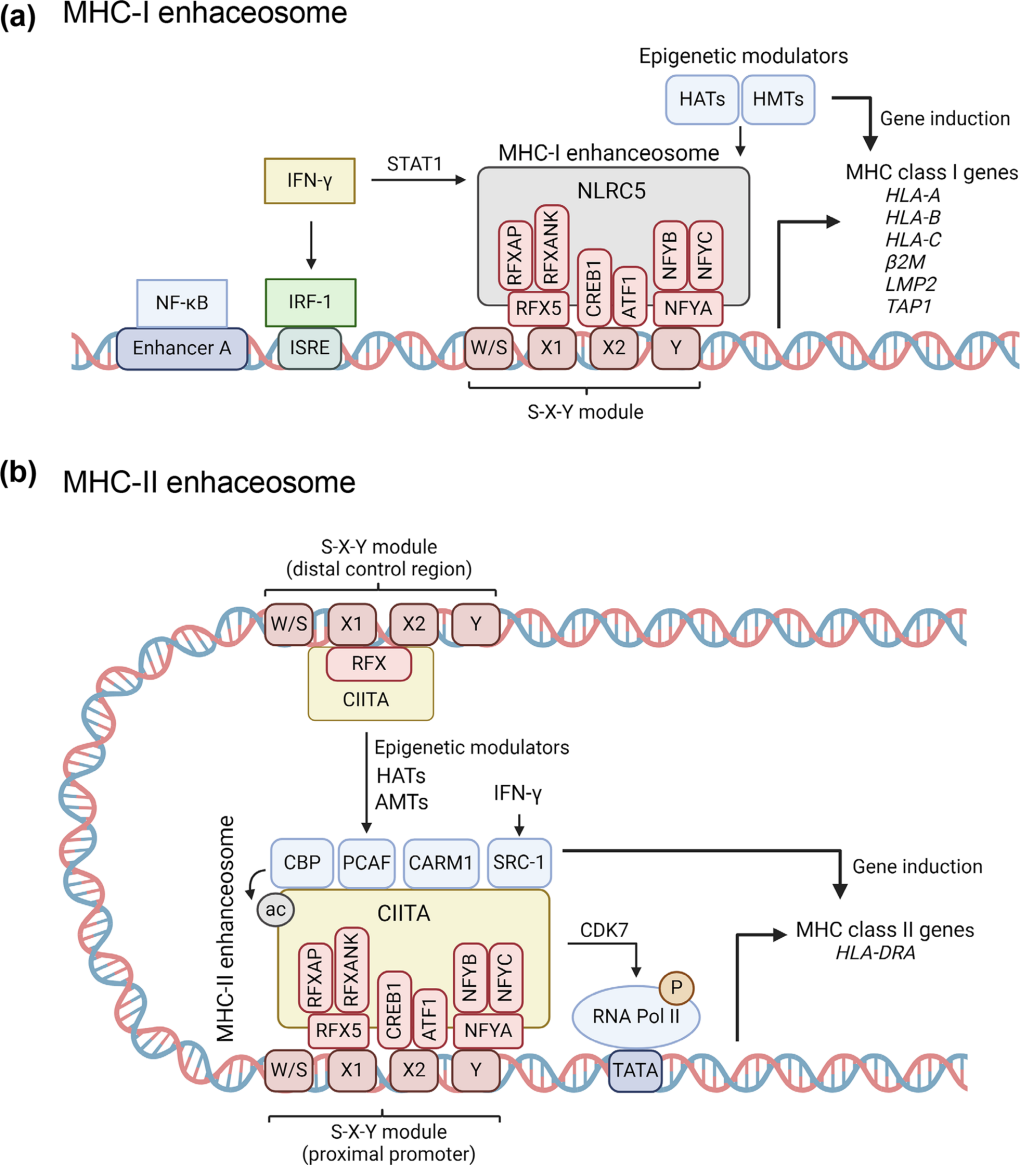
Transcriptional and epigenetic modulation of MHC class I and class II genes by MHC-I/II enhanceosomes. **a** MHC-I enhanceosome recruits HATs and HMTs to MHC-I class I gene promoter regions inducing the expression of MHC-I antigen presentation pathway (APP)-associated genes. **b** MHC-II enhanceosome-induced DNA looping facilitates HAT and AMT recruitment into the proximal promoter of MHC class II genes enabling MHC-II APP-associated gene induction. IFN-γ, interferon-gamma; HAT, histone acetyltransferase; HMT, histone methyltransferase; AMT, arginine methyltransferase; IRF-1, interferon regulatory factor 1; ISRE, interferon-sensitive response element; NF-κB, nuclear factor kappa-light-chain-enhancer of activated B cells; STAT1, signal transducer and activator of transcription 1, CDK7; cyclin-dependent kinase 7; RNA Pol II, RNA polymerase II; TATA, Goldberg–Hogness (TATA) box. Panel (**a**) adapted from Jongsma MLM et al., *Mol Immunol* 2019;113:17 and Panel (**b**) adapted from Masternak K et al., *Genes Dev* 2000;14(9): 1159. Created with BioRender.com on April 24, 2024

Antigen presentation by MHC-I and MHC-II cell surface molecules and the corresponding CD8^+^ and CD4^+^ T cell activation have been reviewed extensively [4, 5]. We focused on the epigenetic regulation of MHC components, including human leukocyte antigen (HLA) factors, in the context of specific gut pathologies. Transcription of MHC-I genes is controlled by conserved *cis*‑acting regulatory elements at the proximal promoters. The promoter generally contains an enhancer A element which contains nuclear factor‑κB (NF‑κB) binding sites, an interferon (IFN)‑stimulated response element (ISRE), and an S-X-Y module. The S-X-Y module consists of four different elements, the W/S, X1, X2 and Y boxes. Specifically, the X1 box binds to the regulatory factor X complex (RFX), which consists of RFX5, RFX‑associated ankyrin‑containing protein (RFXANK) and RFX‑associated protein (RFXAP) [6–10]. The X2 box is bound by cAMP‑responsive element‑binding protein 1 (CREB1) and activating transcription factor 1 (ATF1); whereas the Y box is bound by nuclear transcription factor Y (NFY), which consists of the NFYA, NFYB and NFYC subunits [11–13]. Together, the assembly of these proteins to the S-X-Y module is critical for the transcription of MHC-I genes (Fig. 1a) [2, 14].

However, MHC-I expression also involves NLR Family CARD Domain Containing 5 (NLRC5), as a ‘master’ MHC-I transactivator [2]. The tripartite domain structure in NLRC5 contains an N-terminal caspase activation and recruitment domain (CARD), a centrally located nucleotide-binding domain (NBD) and carboxy‑terminal leucine‑rich repeats (LRRs) [15–18]. Moreover, transcription of *NLRC5* can be efficiently induced by IFN-γ, via signal transducer and activator of transcription 1 (STAT1) interactions with the corresponding gene promoter region [19, 20]. Genome-wide expression profiles in NLRC5 wild-type or mutant human cell lines revealed that NLRC5 was crucial for the efficient induction of MHC-I associated genes. Notably, NLRC5 not only induced the expression of classical MHC-I genes (*HLA‑A*, *HLA‑B* and *HLA‑C*), but also upregulated accessory components, such as the β_2_ Microglobulin gene (*β2M*), *Low-molecular mass protein 2* (*LMP2,* also known as *Proteasome 20S subunit beta 9/PSMB9*) and *Transporter associated with antigen processing 1* (*TAP1*) (Fig. 1a) [15].

The nucleotide-binding domain (NBD) of NLRC5 is essential for nuclear import and MHC-I gene regulation [21]. Although NLRC5 lacks a DNA‑binding domain, it forms a multi-protein complex with RFX, CREB1-ATF1 and NFY known as the MHC enhanceosome, assembled on the S-X-Y module [22]. Nuclear NLRC5 also acts as a platform for histone-modifying enzymes that regulate chromatin dynamics. For example, NLRC5 has synergistic interactions with histone acetyltransferases (HATs) that activate human MHC class I gene transcription and promote histone H3 lysine 27 methylation (H3K9me) at the proximal promoter of *H2-K1*, a murine ortholog of classical human MHC-I genes (Fig. 1a) [23, 24].

The polymorphic MHC-I chain-related proteins A and B (MICA and MICB) are induced by cellular stress or neoplastic transformation [25, 26]. Despite homology with classical MHC class I molecules, MICA and MICB are stably expressed on epithelial cells without association with B2-microglobulin, a key subunit of MHC-I complexes [27]. Recognition by CD8^+^ T cells and NK cells involves the lectin-like NKG2D receptor and plays a role in autoimmunity and tumor recognition [28, 29]. Epigenetic regulation of MIC factors is poorly understood, although recent evidence implicated a role in Merkel cell carcinoma [30].

The discovery of NLRC5 as a key MHC class I transactivator (CITA) paralleled prior research on the corresponding master regulator of MHC-II-dependent genes, known as class II transactivator (CIITA) [31, 32]. Like NLRC5/CITA, CIITA does not bind directly to DNA, but recruits transcription factors and other protein partners that modify chromatin interactions and the local epigenetic landscape [31, 32]. The presence of an S-X-Y module enables interacting proteins, similar to those on MHC-I promoters, to form an MHC-II enhanceosome that acts as a scaffold to recruit CIITA [31, 32]. A regulatory module similar to the X–Y motif is located upstream of some MHC-II genes, such as *HLA-DRA*. After CIITA binds to the MHC-II enhanceosome, DNA looping facilitates synergistic interactions with chromatin modifiers and HATs, such as CREB binding protein (CBP), P300/CBP-associated factor (PCAF), and steroid receptor coactivator-1 (SRC-1) [33]. Thus, CIITA can modify the chromatin landscape from a repressive ‘closed’ state to an open conformation, allowing for other DNA-binding proteins to interact with MHC-II promoters (Fig. 1b).

Interestingly, CIITA is a direct target for acetylation by CBP and PCAF, a process that can influence CIITA nuclear translocation [34]. Other HATs, such as SRC-1, are recruited to MHC-II promoters only after IFN-γ stimulation and help to dampen estradiol-mediated inhibition of MHC-II expression (Fig. 1b) [35]. Chromatin immunoprecipitation (ChIP) in cell lines that are responsive to IFN-γ established the correlation between CIITA and increased levels of histone H3 and H4 acetylation, synonymous with CIITA promoting an ‘open’ chromatin state [33]. One report [36] proposed that CIITA might possess HAT activity, although this awaits independent corroboration.

As counterpoint to HATs, cellular histone deacetylases 1 and 2 (HDAC1, HDAC2) repress MHC-II gene expression in a broad range of cancers [37]. The HDACs and their corepressors, such as mSin3A, trigger enhanceosome disassembly and override IFN-γ-mediated upregulation of MHC-II genes. In cancer immunotherapy, HDAC inhibitors can increase antitumor immunity by facilitating histone acetylation near MHC-II promoters [33, 37]. Other post-transcriptional modifications, such as mono-ubiquitination, regulate the function of CIITA leading to increased gene activation [38]. Additionally, CIITA can associate with coactivator-associated arginine methyltransferase 1 (CARM1), leading to arginine methylation on histone H3R17 and CBP, enhancing MHC-II promoter interactions and MHC-II gene expression [39]. Moreover, CIITA can promote cyclin-dependent kinase 7-mediated phosphorylation of RNA polymerase II, directly initiating the synthesis of mRNA transcripts from the corresponding genes (Fig. 1b) [33, 40].

Unlike MHC-I complexes which are present in all mammalian cells, MHC-II complexes are expressed mainly in antigen-presenting cells, such as dendric cells, macrophages or B cells. However, as previously noted, IFN-γ induces MHC-II expression in a CIITA-dependent manner in other cell types [41]. Among the four *CIITA* promoter regions, designated as PI-PIV, IFN-γ acts mainly through *CIITA-PIV*. Distinct cell types, such as trophoblasts and tumor cells, have mechanisms to diminish the IFN-γ response acting though *CIITA-PIV* [42]. One such mechanism is DNA hypermethylation at CpG islands around *CIITA-PIV*, which decreases transcription factor binding [42, 43]. Studies with DNA methyltransferase (DNMT) inhibitors such as 5-azacytidine (5-AZA), plus HDAC inhibitors such as Trichostatin A (TSA), established that epigenetic mechanisms silence *CIITA-PIV* in tumor cells, and that interference with such mechanisms restores CIITA expression [42, 44, 45]. Thus, downregulation of CIITA and MHC-II-associated gene expression may be a common mechanism of immune escape in cancer cells. Accordingly, studies in uveal melanoma cells observed that treatment with 5-AZA restored the IFN-γ-mediated inducibility of CIITA.

Certain gastric and colorectal cancers do not respond to IFN-γ due to DNA hypermethylation near *CIITA-PIV* [42, 46]. Histone H3K9 methylation was present as a gene-silencing mark, suggesting that selective inhibition of the corresponding H3K9 methyltransferase might be a therapeutic approach to recover CIITA expression [47]. Collectively, these findings highlight the critical importance of CIITA as a master regulator of MHC-II-associated gene expression, its deregulation in cancer cells, and the contributions of epigenetic mechanisms to pathogenesis.

## Immunoepigenetics and the tumor microenvironment

Epigenetic alterations in the tumor microenvironment (TME) affect the dynamic crosstalk between cancer and immune cells exhibiting differential expression of MHC antigen presentation pathway (APP) components. Immune cell subtypes include tumor-associated macrophages (TAMs), neutrophils, natural killer cells, T and B lymphocytes, as well as fibroblasts, stromal cells, and blood vessels [48–50]. The TME can either inhibit or promote tumorigenesis and lead to chemotherapy resistance, with implications for cancer development and treatment [51–54]. For example, a member of the tumor necrosis factor receptor superfamily, Decoy receptor 3 (DcR3), was reported to be overexpressed in pancreatic cancer and other malignancies [49]. DcR3 induced TME dendritic cell apoptosis and TAM differentiation into an M2 phenotype, which was associated with suppression of inflammatory mechanisms and increased angiogenesis, leading to tumor progression [49, 50]. Additionally, DcR3 diminished the expression of genes associated with the MHC-II APP, including classical (*HLA-DR, -DP* and -*DQ*) and non-classical (*HLA-DM* and *-DO*) MHC-II genes, as well as *CD74* and *CIITA*, in monocyte-derived macrophages (MDMs) [49]. The reduced antigen-presenting capacity in TAMs and T cell anergy diminished the host immune response and promoted cancer immune evasion. Moreover, extracellular signal-regulated kinase/c-Jun N-terminal kinase-associated histone deacetylation has been identified as a mechanism for DcR3-mediated MHC-II suppression [49, 50, 55]. Global histone deacetylation affected all *CIITA* promoters after MDMs were treated with DcR3, implicating chromatin remodeling and alterations in HDAC/HAT expression, activity, or recruitment; however, the precise mechanisms remain to be elucidated [49, 50]. DcR3 also regulated TAM differentiation in murine models, with increased arginase activity and decreased MHC-II complexes. Downregulation of MHC-II enhanced tumor growth and impaired tumor-associated antigen presentation to T cells, dampening the anti-tumoral adaptive immune response [49, 56]. The HDAC inhibitor sodium valproate/valproic acid (VPA) partially restored MHC-II expression and circumvented the enhanced tumor growth in DcR3-transgenic mice inoculated subcutaneously with murine colon adenocarcinoma cells, indicating that epigenetic modulation of TAM differentiation and MHC-II expression had a crucial role in DcR3-driven tumor progression [56, 57]. The clinical relevance of VPA as an anticancer therapeutic agent is well established, supporting HDAC inhibition for APP enhancement, either as a single agent or by augmenting standard of care in patients with tumors expressing high DcR3 levels, such as pancreatic cancer [56, 58].

## Gastric *cancer* and immunoepigenetic deregulation

**Fig. 2:**
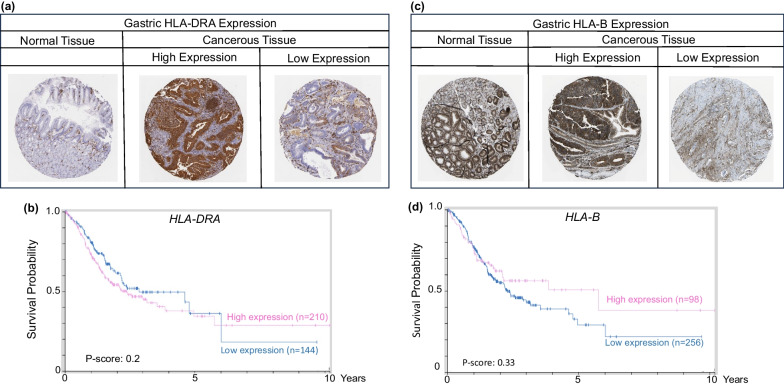
MHC-I and MHC-II expression in gastric normal and cancer tissues. **a**, **c** Heterogeneous expression of HLA-DRA and HLA-B proteins in human tissue microarrays (TMAs). **b**, **d** Kaplan–Meier (KM) curves for *HLA-DRA* and *HLA-B* gene expression in gastric cancer. For quantification of percent labeling in TMAs see https://www.proteinatlas.org/

Heterogeneous HLA-DRA protein expression was observed in tissue microarrays of human gastric cancer (Fig. 2a), with the Kaplan–Meier survival curves, obtained from The Human Protein Atlas, indicating a non-statistically significant difference for high *vs.* low *HLA-DRA* expression of the α subunit of HLA-DR (Fig. 2b P-score = 0.20). In human gastric cancer cell lines, expression of HLA-DR in response to IFN-γ was dampened due to *CIITA-PIV* hypermethylation [59]. Meazza et al. noted that the upregulation of *HLA-DR* after introduction of exogenous CIITA led to T cell activation and tumor rejection, suggesting that CIITA could be a valuable target for future therapeutics [60]. Satoh et al*.* confirmed that 14 of 20 gastric and colon cancer cell lines expressed *HLA-DR* after IFN-γ stimulation, and that exogenous CIITA in cell lines that lacked HLA-DR prompted re-expression of the target gene, with and without IFN-γ stimulation. Subsequent work noted that CIITA was expressed only when *CIITA-PIV* was unmethylated [59]. It was confirmed that *CIITA-PIV* had a CpG island that was methylated close to the transcription start site, and also involved histone deacetylation in the 5’ region of the gene. Supporting the dynamic crosstalk between epigenetic regulatory mechanisms, IFN-γ increased histone acetylation more effectively in gastric cell lines harboring unmethylated CpG sites in the *CIIT-PIV* region, [59].

In a similar fashion to HLA-DRA*,* heterogeneous protein expression was detected for classical MHC-I players, such as HLA-B (Fig. 2c), and survival in patients beyond the 2-year timepoint tended to be improved by high *HLA-B* levels (Fig. 2d). Downregulation of HLA-A/B/C has been associated with promoter methylation of the corresponding genes in other types of cancer, such as melanoma and neuroblastoma, compared to adjacent non-tumor tissues, suggesting that MHC-I surface complexes could be restored in gastric cancer by DNA methyltransferase inhibitors [61]. These findings re-affirm the integration of reversible epigenetic mechanisms, such as DNA methylation and histone acetylation, and provide avenues for HDAC + DNMT combinatorial therapies in gastric cancer interception.

## Pancreatic *cancer* and immunoepigenetic deregulation

**Fig. 3:**
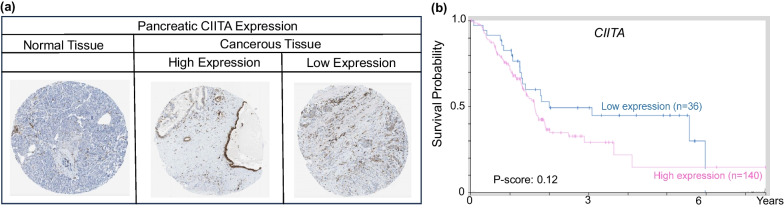
MHC-II-related expression in pancreatic normal and cancer tissues. **a** Weak-to-moderate CIITA protein expression in human TMAs. **b** KM survival curves for CIITA gene expression in pancreatic cancer. For quantification of percent labeling in TMAs see https://www.proteinatlas.org/

Pancreatic cancer is another malignancy that is influenced by DNA methylation and gene silencing [62–66]. Studies by Cao et al*.* in a human pancreatic cancer cell line of ductal origin (PANC-1) revealed *CIITA* promoter hypermethylation and low MHC-II expression, even after stimulation with IFN-γ. Methylation of *CIITA* was decreased after combined treatment with IFN-γ, 5-AZA and the pan-HDAC inhibitor suberoylanalide hydroxamic acid (SAHA) [67–69]. In PANC-1 cells, higher doses of 5-AZA caused complete demethylation of the *CIITA* promoter, reflected by increased *CIITA* and *MHC-II*-associated gene expression [67]. Interestingly, a vaccine was developed using PANC-1 cells with augmented MHC-II expression following epigenetic drug combinations. In murine models, the vaccine enhanced lymphocyte proliferation, CD8^+^ T cell anti-tumoral activity, and IFN-γ plus IL-2 secretion by Th1-type lymphocytes, while decreasing TGF-β and IL-4 secretion by Th2-type lymphocytes. These findings indicated a shift in the Th1/Th2 ratio, increasing the number of Th1-type cells and resulting in increased activity of cytotoxic T cells [67]. Thus, vaccines produced by a combination of HDAC plus DNMT inhibition could be an approach for pancreatic cancer interception, increasing MHC-II expression and triggering an anti-tumoral immune response. Tissue microarrays showed uniform low levels of CIITA protein in pancreatic cancers, with focal expression (Fig. 3a). Surprisingly, patients with high *CIITA* levels tended to have worse prognosis, although this was not statistically significant due to the low sample number (Fig. 3b, Pscore = 0.12).

## Colorectal *cancer* and immunoepigenetic deregulation

In Colon 26 murine colon adenocarcinoma cells, IFN-γ induced transcription from the *CIITA-PIV* locus, but not from other *CIITA* promoters, whereas TSA treatment induced *CIITA-PIII* and not *CIITA-PIV* [70]. Transfection of Colon 26 cells with a dominant negative CIITA plasmid did not inhibit TSA-mediated MHC-II expression, while IFN-γ-mediated MHC-II expression was impeded [54, 70]. Thus, even in a single murine colon cancer cell line, pathways converge that are CIITA-dependent and CIITA-independent. Furthermore, IFN-γ but not TSA increased protein levels of IRF-1 and phosphorylation of STAT-1, which are essential for *CIITA-IV* activation [70–72]. Combinatorial treatment of IFN-γ with TSA, or the subsequent addition of IFN-γ after TSA treatment, further increased CIITA induction when compared to IFN-γ alone. Interestingly, pretreatment with TSA followed by IFN-γ had the opposite effect, showing repression of total CIITA and *CIITA-PIV* [37, 70]. This unexpected finding was associated with a decrease in STAT-1α phosphorylation in Colon 26 cells pretreated with TSA. These findings indicate that HDAC inhibition can increase or decrease MHC-I expression in a context-dependent manner [70].

Although TSA increased histone acetylation and induced MHC-II-dependent gene expression, the precise mechanisms are not well understood [37, 70, 73]. As a consequence of TSA-mediated changes in histone acetylation, increased H3K4 methylation and decreased H3K9 methylation was observed, which are hallmarks of enhanced gene expression [70, 74]. The H3K9 methylation mark provided a docking site for heterochromatin protein-1 (HP-1) in the promoter region of *MHC-II* genes, leading to localized gene silencing; thus, decreased H3K9 methylation after TSA treatment interfered with the repressive actions HP-1 [70, 75]. A TSA-mediated shift between HAT and HDAC activities also affected post-translational modifications on nonhistone proteins, including transcription factors that promote MHC-II expression. For example, Greer et al*.* observed that CBP/p300 HATs and TSA-mediated HDAC inhibition promoted CIITA monoubiquitylation and enhanced MHC-II expression [70, 76]. However, further studies are needed to determine which mechanism(s) are critical for upregulation of *CIITA* in Colon 26 cells.

Another approach might be to combine HDAC inhibition with standard of care chemotherapy [77]. For example, the HDAC inhibitor depsipeptide (Dep) enhanced the antitumor efficacy in human colon cancer cells of a first line drug used in the treatment of colorectal cancer, namely 5-fluorouracil (5-FU) [78]. Dep + 5-FU reduced colony formation and cell viability, and increased caspase 3/7 activation in HCT116 cells, compared to 5-FU monotherapy. Microarrays revealed upregulation of genes associated with apoptosis and cell death, as well as MHC-II classical genes such as *HLA-DPB1, HLA-DQB1, HLA-DRA* and *HLA-DRB1* [82]. Other colon cancer cell lines were less responsive to Dep + 5-FU, revealing a modest increase in *HLA-DRA,* and none of the cell lines exhibited changes in MHC-I genes. In HCT116 cells, increased CIITA, CREB3/5 and PCAF after Dep + 5-FU treatment was observed [78]. As previously established, CREB3 and CREB5 participate in the positive regulation of MHC-II genes, whereas PCAF has HAT activity and when associated with CIITA enhances apoptosis [31, 78, 79].

A colon cancer cell line lacking DNMT1 and DNMT3b exhibited reduced methylation on *CIITA-PIV*, implicating a role for DNMTs in *CIITA* regulation [59, 80]. However, *CIITA* expression occurred only after IFN-γ stimulation, due to transcription factor recruitment to unmethylated *CIITA-PIV* [59]. Thus, epigenetic modulation of *CIITA-PIV* by DNMT inhibitors might be a therapeutic approach to enhance immunotherapy. For example, combining the DNTM1/DNMT3a inhibitor hydralazine with VPA increased the expression of MICA and MICB ligands on NK cells and induced NK cell cytotoxicity against tumor cells. This effect was related to the presence of histone H3K4me2 at the *MICA* and *MICB* promoters [81]. These studies illustrated how MICA and MICB expression is mostly controlled by DNA methylation, further highlighting the relevance that DNMT inhibitors could have in a subset of colorectal cancer.

**Fig. 4:**
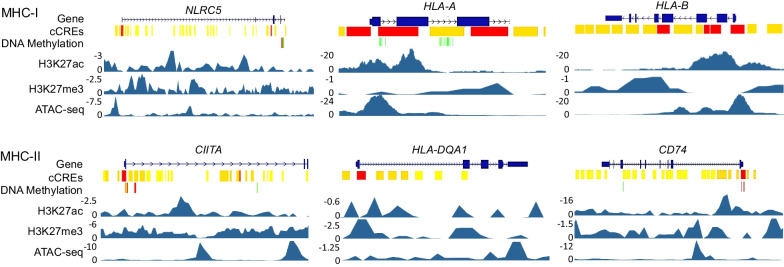
Epigenetic landscapes and chromatin accessibility of MHC-I and MHC-II genes in human colon cancer cells. Chromatin immunoprecipitation sequencing (ChIP-seq) in HCT116 cells for histone H3K27ac and H3K27me3, or reduced-representation bisulfite sequencing for DNA methylation, with color coding representing methylated CpG island density (fold-change vs. normal). cCREs, cis-regulatory elements (red promoter-like; orange proximal enhancer-like; yellow distal enhancer-like); ATAC-seq, assay for transposase-accessible chromatin with sequencing. Data from the UCSC Genome Browser (https://genome.ucsc.edu) and ENCODE (https://www.encodeproject.org)

**Fig. 5:**
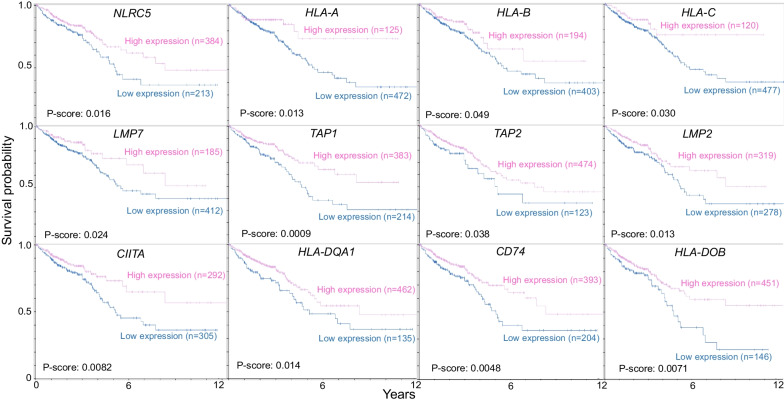
KM survival curves for colorectal cancer patients with high *vs.* low MHC-I (top and middle panels) and MHC-II gene expression (lower panels). Data obtained from the Human Protein Atlas (https://www.proteinatlas.org/)

**Fig. 6:**
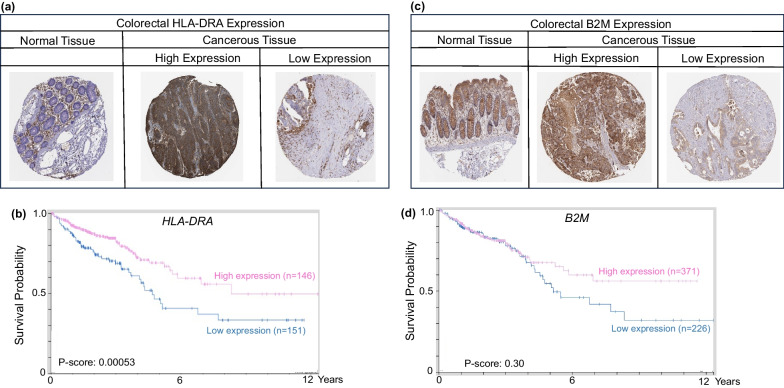
MHC-I and MHC-II expression in colorectal normal and cancer tissues. **a**, **c** Heterogeneous expression of HLA-DRA and B2M proteins in human TMAs. **b**, **d** KM survival curves for *HLA-DRA* and *B2M* gene expression in colorectal cancer. For quantification of percent labeling in TMAs see https://www.proteinatlas.org/

On the other hand, metabolomic data from dietary spinach intake in the Apc-mutant polyposis in rat colon (Pirc) model implicated linoleate and butanoate metabolites that targeted HDAC activity and IFN-γ signaling [82]. Mechanistic studies with 13(S)-hydroxyoctadecadienoic acid (13S-HODE) and (S)-2-hydroxybutanoate (S-2HB) revealed elevated β2m protein levels, increased cell surface β2m expression, and induced IL-2 secretion from T cell hybridoma co-cultures, consistent with enhanced MHC-I cell surface presentation on colon cancer cells [82]. Epigenetic and chromatin accessibility marks implicated in the etiology of human colon cancer, obtained from UCSC Genome Browser and ENCODE databases, identified histone acetylation/methylation and DNA methylation, in the regulation of MHC-I and MHC-II gene expression (Fig. 4). For an entire suite of MHC-I and MHC-II genes, low expression in human colorectal cancer was synonymous with worse survival (Fig. 5). In tissue microarrays from colorectal patients, a broad range of MHC-I and MHC-II protein expression was detected, as exemplified for B2M and HLA-DRA, respectively (Fig. 6a, c). Kaplan–Meier curves (Fig. 5, Figure b,d) supported the hypothesis that epigenetic-targeted interventions that re-express MHC factors could enhance survival, and be valuable for immune interception in a subset of colorectal cancer patients.

## Esophageal *cancer* and immunoepigenetic deregulation

Epigenetic modifications also play a critical role in the progression of upper GI malignancies [83]. Kazakh esophageal squamous cell carcinoma (ESCC) exhibited unusually high levels of *HLA-DQ, HLA-DR* and *HLA-DP*, whereas later clinical stages had reduced expression of the corresponding genes, indicating that decreased MHC-II molecules facilitated immune escape and tumor progression [84]. Hu et al*.* demonstrated that *HLA-DRB1* methylation levels were lower in Kazakh ESCC when compared to normal tissue, whereas methylation levels of *HLA-DQB1* were increased in tumor samples. The methylation status of *HLA-DRB1* might promote tumor progression, as evidenced by *HLA-DRB1* CpG16 hypermethylation and subsequent gene silencing in later clinical stages and vice versa for *HLA-DQB1*, where lower methylation levels of CpG16-17 were associated with increased aggressiveness of Kazakh ESCC [84]. These findings suggested that changes in the promoter methylation status of *HLA-DRB1* and *HLA-DQB1* are tightly associated with Kazakh ESCC progression and could serve as cancer biomarkers. Because *HLA* genes can harbor differential methylation profiles in Kazakh ESCC, and DNA methyltransferase inhibitors would likely reverse the hypomethylation status of both *HLA-DRB1* and *HLA-DQB1*, it would be important to sub-group ESCC patients before adopting this kind of ‘precision’ epigenetic therapy.

**Fig. 7:**
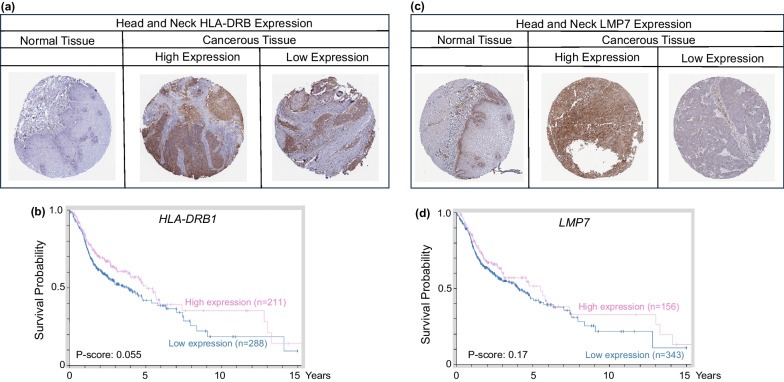
MHC-I and MHC-II expression in head and neck cancer and normal tissues. **a**, **c** Heterogeneous expression of *HLA-DRB1* and *LMP7* proteins in human TMAs. **b**, **d** KM survival curves for *HLA-DRB1* and *LMP7* gene expression in head and neck cancer. For quantification of percent labeling in TMAs see https://www.proteinatlas.org/

In the case of MHC-I genes, downregulation of HLA-A and β2M has been previously linked to esophageal carcinoma pathogenesis, while overexpression of microRNAs, miR-148a-3p and miR-125a-5p, has been associated with downregulation of MHC-I genes, such as *TAP2,* in this type of malignancies [85]. Moreover, multiple CpG sites were linked to loss of *HLA-B* and other associated genes, such as *TAP2* and *LMP7* [86]. However, human tissue microarrays indicated that patients with head and neck cancer exhibited heterogenous expression of HLA-DRB and LMP7 proteins (Fig. 7a, c), and the corresponding gene expression changes did not predict a significant difference in survival probability [86]. (Fig. 7b, d).

## Liver *cancer* and immunoepigenetic deregulation

**Fig. 8:**
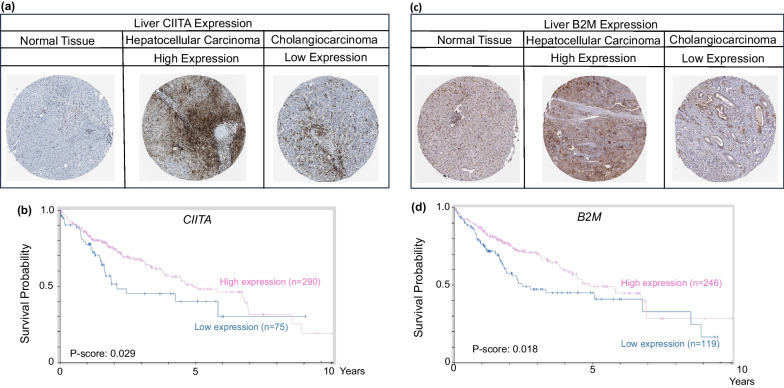
MHC-I and MHC-II expression in hepatic normal and cancer tissues. **a**, **c** Heterogeneous expression of CIITA and B2M proteins in human TMAs. **b**, **d** KM survival curves for CIITA and B2M gene expression in liver cancer. For quantification of percent labeling in TMAs see https://www.proteinatlas.org/

Vorinostat first exhibited promise as an HDAC inhibitor and anticancer agent in patients with advanced cutaneous T cell lymphoma [87, 88], followed by malignancies such as hepatocellular carcinoma [30, 89, 90]. Vorinostat downregulated microRNA-20a, diminished STAT3 tyrosine phosphorylation, and increased histone acetylation at *MICA* and *MICB* promoters, leading to increased expression of these genes and enhanced natural killer (NK) cell-mediated tumor targeting [90]. The HDAC inhibitor MS-275 epigenetically modified exosomes secreted by human liver cancer cells, increasing the expression of MICA, MICB, and heat shock protein 70, and enhancing the cytotoxic effects of NK cells [91]. These data indicated that *MICA* and *MICB* expression in liver cancer is regulated by histone acetylation, and that HDAC inhibitors might provide for targeted therapies. Representative examples of MHC-I and MHC-II players in liver cancer, such as B2M and CIITA, showed similar patterns as other cancer types, with heterogenous protein expression in tissue microarrays (Fig. 8a, c) and low gene expression being predictive of poor survival (Fig. 8b, d). Tumor subtype was an additional variable, with hepatocellular carcinoma having high expression and cholangiocarcinoma having low CIITA and B2M protein levels (Fig. 8a, c).

## Conclusions

Demographic and epidemiologic data indicate that the burden of GI cancers and other malignancies remains a major health concern in the US and worldwide [92, 93]. For example, a 50% increase in death from colon cancer and a doubling of rectal cancer mortality is predicted in the US by 2035 [94]. Similarly, new gastric cancer cases diagnosed per year are expected to increase from 1.09 to 1.77 million worldwide from 2020 to 2040, representing a 62% increase in incidence [95]. In this same timeframe, esophageal cancer incidence and mortality are predicted to rise 58.1% and 61.7% worldwide, respectively [96]. Thus, there is an urgent need for new and improved approaches to cancer interception. The reversibility of deregulated epigenetic landscapes in numerous malignances provides an attractive avenue for GI cancer intervention [97]. Understanding the fundamental epigenetic mechanisms that govern the expression of MHC-I, MHC-II, and MHC-associated molecules is an area of growing interest, especially in the field of immunotherapy, via epigenetic monotherapies or as adjuvants to standard of care. Drugs that modify distinct epigenetic processes, such as DNA methylation or histone post-translational modifications, can upregulate MHC-associated molecules in GI malignancies, suggesting the potential to re-engage the host immune system for enhanced cancer immune evasion. It is likely that refinements will be made to the current arsenal of ‘epigenetic’ drugs, to improve efficacy while reducing toxicity. This might involve patient-specific molecular phenotyping combined with the inhibition of selected epigenetic ‘readers’, ‘writers’ and ‘erasers’ [98–100]. For example, histone methylation inhibitors targeting the polycomb repressive complex 2 (PRC2) have shown promise in enhancing cancer cell immunogenicity through MHC upregulation [101, 102]. Notably, PRC2 inhibitors are in clinical trials for the treatment of lymphoma and nasopharyngeal carcinoma, and an orally bioavailable PRC2 inhibitor was recently approved for epithelioid sarcoma [103, 104]. Additionally, disrupting the scaffolding capabilities of other histone methyltransferase complexes, through the use of WD repeat-containing protein 5 (WDR5) inhibitors, demonstrated antitumor efficacy in preclinical models of colon cancer [105]. These developments suggest that histone methylation inhibitors will have clinical utility for MHC re-expression, and that drug repurposing could facilitate immunomodulatory targeting of GI malignancies for which altered histone methylation has been implicated [106].

**Fig. 9:**
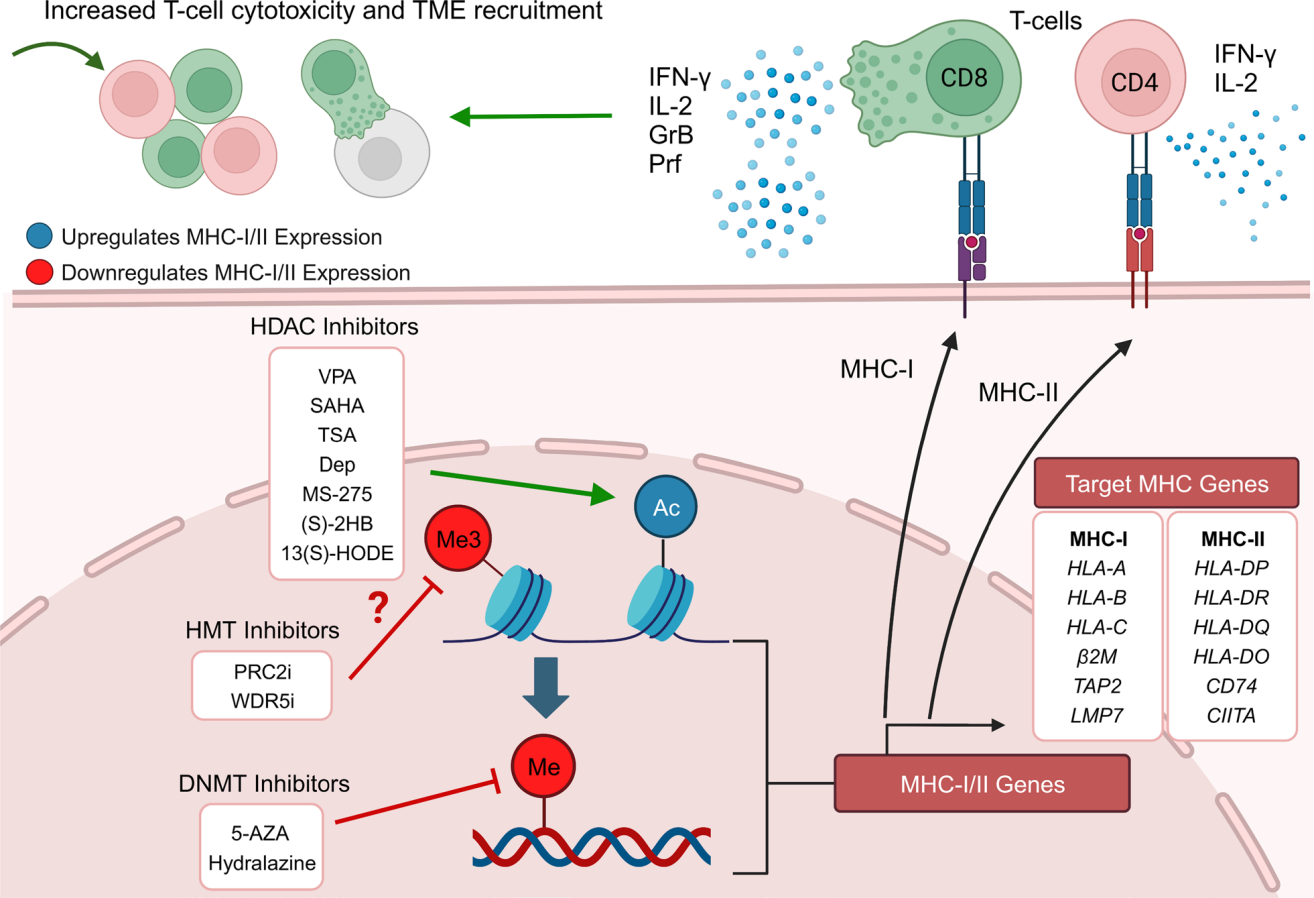
Epigenetic Regulation of MHC-I and MHC-II in gastrointestinal cancer. Epigenetic mechanisms and drugs affecting MHC-I and MHC-II expression in gastrointestinal cancer cells, including histone deacetylase (HDAC) and DNA methyltransferase (DNMT) inhibitors, as well as the possible role of histone methyltransferase (HMT) inhibitors. IFN-γ, interferon-gamma; IL-2, interleukin-2; GrB, Granzyme B; Prf, Perforin; PRC2i, PRC2 inhibitors; WDR5i, WDR5 inhibitors. Created with BioRender.com

As previously mentioned, MHC upregulation could be a critical mechanism to augment the efficacy of current immunotherapeutic strategies. For example, Birinapant, a mimetic of the second mitochondrial-derived activator of caspases (SMAC), was able to upregulate MHC-I in preclinical melanoma models, which resulted in cancer cell sensitization to CD8^+^ T cell-dependent cytotoxicity and enhanced immune checkpoint blockade efficacy [107]. Another study demonstrated that MHC-I was upregulated by inhibition of the deubiquitinase USP8, through activation of the NF-κB pathway. USP8 inhibition in combination with PD-1/PD-L1 blockade increased CD8^+^ T cell infiltration and diminished tumor growth, compared to immune checkpoint blockade monotherapy. This drug combination resulted in improved survival using various preclinical murine tumor models, including mouse colon adenocarcinoma allografts, indicating that findings could be generalized to GI malignancies [108]. Additionally, recent publications examined the role of NLRC5 and MHC-I molecules in cancer immunotherapy sensitivity [109, 110]. These reports shed light on how NLRC5 upregulation and the subsequent increase of MHC-I associated proteins, through the use of epigenetic modifiers such as DNA and histone methylation inhibitors, could reverse unresponsiveness to immunotherapy. Collectively, the findings support the potential use of PRC2 inhibitors and other epigenetic drugs as immunotherapeutic or preventive agents for various GI malignancies. As the field progresses and evolves, new avenues are anticipated for human translation and immunoepigenetic cancer interception via MHC gene upregulation [111–114]. Pertinent information from the systematic review is summarized in Table 1 and Fig. 9; survival data were obtained from The Human Protein Atlas (Figs. 2, 3, 5, 6, 7, 8) while epigenetic landscape and chromatin accessibility data were attained from the UCSC Genome Browser and the ENCODE database (Fig. 4), as indicated in each figure legend [115–117].

**Table 1:** Epigenetic drugs and their effects on MHC-I/MHC-II expression in GI malignancies

Drugs	Mechanism	Malignancy	Outcomes	References
Sodium Valproate (VPA)	HDAC class I inhibitor	Colorectal	Partially restored MHC-II expression and decreased tumor growth of murine colorectal carcinoma cells in DcR3 transgenic mice	PMID:22287720
5-Azacytidine (5-AZA)	DNMT inhibitor	Pancreatic	Decreased *CIITA* methylation after combined treatment (5-AZA + SAHA + IFN-γ) in a human pancreatic cancer cell line of ductal origin (PANC-1) increased MHC-II mRNA levels	PMID:25198660
Vorinostat/Suberoylanilide hydroxamic Acid (SAHA)	Pan-HDAC inhibitor	Pancreatic		
		Liver	Upregulation of *MICA* and *MICB* expression by increasing histone acetylation in the chromatin-associated regions of these genes, thus enhancing the sensitivity of hepatocellular carcinoma to NK cell-mediated lysis	PMID: 25393367
Trichostatin A (TSA)	HDAC class I and II inhibitor	Colorectal	Increased MHC-II expression in Colon 26, a murine colon adenocarcinoma cell line, inducing *Ciita-PIII* and also via a CIITA-independent mechanism	PMID:16120330
Depsipeptide (Dep)	HDAC class I inhibitor	Colorectal	Dep + 5-FU exhibited antitumor efficacy in HCT116 human colon cancer cells by reducing colony formation/cell viability and upregulating expression *CIITA* and other MHC-II genes	PMID:27509880
Hydralazine	DNMT1 and DNMT3a inhibitor	Colorectal	Hydralazine + VPA increased *MICA* and *MICB* expression in NK cells, inducing NK cell cytotoxicity against tumor cells, via histone activation marks at *MICA* and *MICB* promoters	PMID:21805029 PMID:26942461
MS-275	HDAC class I inhibitor	Liver	Epigenetically modified exosomes secreted by HepG2 cells, a human liver cancer cell line, increased *MICA* and *MICB* expression and enhanced the cytotoxic effects of NK cells	PMID:24359553
13(S)-hydroxyoctadeca- dienoic acid (13(S)-HODE)	HDAC1 and HDAC3 inhibition	Colorectal	Augmented cell surface β2m expression, resulting in increased IL-2 secretion from T cell CD8^+^ hybridomas when co-cultured with murine colon cancer cells, indicative of increased MHC-I cell surface presentation	PMID: 35159382
(S)-2-hydroxybutanoate ((S)–2HB)				

Epigenetic drugs, their mechanisms of action, target organs and effects regulating MHC-I or MHC-II expression in GI cancers. HDAC, Histone deacetylases; DNMT, DNA methyltransferases

## Data Availability

The datasets analyzed during the current study are publicly available online as follows: The Human Protein Atlas (https://www.proteinatlas.org/), UCSC Genome Browser (https://genome.ucsc.edu/), and ENCODE (https://www.encodeproject.org/). Datasets were accessed as indicated in the text and figures.

## Abbreviations

DNA: Deoxyribonucleic acid
MHC: Major histocompatibility complex
RNA: Ribonucleic acid
GI: Gastrointestinal
CD8^+^: Cluster of differentiation 8-positive T cells
CD4^+^: Cluster of differentiation 4-positive T cells
HLA: Human leukocyte antigen
NF-κB: Nuclear factor-kappa B
IFN: Interferon
ISRE: Interferon-stimulated response element
S-X-Y: Elements in MHC-I gene promoter
RFX: Regulatory factor X complex
RFX5: Regulatory factor X 5
RFXANK: Regulatory factor X-associated ankyrin-containing protein
RFXAP: Regulatory factor X-associated protein
CREB1: CAMP-responsive-element-binding protein 1
ATF1: Activating transcription factor 1
NFY: Nuclear transcription factor Y
NLRC5: NLR family CARD domain containing 5
CARD: Caspase activation and recruitment domain
NBD: Nucleotide-binding domain
LRRs: Leucine-rich repeats
IFN-γ: Interferon-gamma
STAT1: Signal transducer and activator of transcription 1
MHC-I: Major histocompatibility complex class I
β2M: Beta-2 microglobulin
LMP2: Low-molecular mass protein 2
PSMB9: Proteasome 20S subunit beta 9
TAP1/2: Transporter associated with antigen processing 1/2
HATs: Histone acetyltransferases
H3K9me: Histone H3 lysine 9 methylation
MICA: MHC-I chain-related protein A
MICB: MHC-I chain-related protein B
NK cells: Natural killer cells
NKG2D: Natural killer group 2, member D receptor
CIITA: Class II transactivator
MHC-II: Major histocompatibility complex class II
S-X-Y: Elements in MHC-II gene promoter
CBP: CREB binding protein
PCAF: P300/CBP-associated factor
SRC-1: Steroid receptor coactivator-1
ChIP: Chromatin immunoprecipitation
HDACs: Histone deacetylases
mSin3A: Mammalian switch-independent 3A
PI-PIV: CIITA promoter regions designated as PI-PIV
CpG: Cytosine-phosphate-guanine
DNMT: DNA methyltransferase
TSA: Trichostatin A
H3K9: Histone H3 lysine 9
TAMs: Tumor-associated macrophages
TME: Tumor microenvironment
T cells: T lymphocytes
B cells: B lymphocytes
DcR3: Decoy receptor 3
TME: Tumor microenvironment
M2: Alternatively activated macrophages
APP: Antigen presentation pathway
HLA-DR: Human leukocyte antigen-DR isotype
HLA-DP: Human leukocyte antigen-DP isotype
HLA-DQ: Human leukocyte antigen-DQ isotype
HLA-DM: Human leukocyte antigen-DM
HLA-DO: Human leukocyte antigen-DO
CD74: Cluster of differentiation 74
MDMs: Monocyte-derived macrophages
VPA: Valproic acid
HLA-DRA: Human leukocyte antigen-DR alpha subunit
HLA-B: Human leukocyte antigen-B isotype
HDAC + DNMT: Histone deacetylase and DNA methyltransferase
SAHA: Suberoylanilide hydroxamic acid
IL-2: Interleukin-2
Th1/Th2: T-helper 1/T-helper 2 cells
TGF-β: Transforming growth factor-beta
IL-4: Interleukin-4
TSA: Trichostatin A
IRF-1: Interferon regulatory factor 1
H3K4: Histone H3 lysine 4
HP-1: Heterochromatin protein-1
5-FU: 5-Fluorouracil
Dep: Depsipeptide
CREB3/5: CAMP-responsive element-binding protein 3 and 5
PCAF: P300/CBP-associated factor
DNMT1: DNA methyltransferase 1
DNMT3b: DNA methyltransferase 3b
PRC2: Polycomb repressive complex 2
ESCC: Esophageal squamous cell carcinoma
LMP7: Low-molecular mass protein 7
FDA: Food and drug administration
STAT3: Signal transducer and activator of transcription 3
MS-275: Entinostat
WDR5: WD repeat-containing protein 5
PD-1: Programmed cell death protein 1
PD-L1: Programmed death-ligand 1
